# Cremation weights for an Italian contemporary sample

**DOI:** 10.1111/1556-4029.70181

**Published:** 2025-09-29

**Authors:** Barbara Bertoglio, Matteo Di Maso, Debora Mazzarelli, Francesca Magli, Alessandra Mazzucchi, Michela Zana, Giulia Caccia, Cristina Cattaneo

**Affiliations:** ^1^ Dipartimento di Sanità Pubblica, Medicina Sperimentale e Forense, Unità di Medicina Legale e Scienze Forensi Università Degli Studi di Pavia Pavia Italy; ^2^ LABANOF, Dipartimento di Scienze Biomediche per la Salute, Sezione di Medicina Legale Università Degli Studi di Milano Milan Italy; ^3^ Dipartimento di Scienze Cliniche e di Comunità Università Degli Studi di Milano Milan Italy

**Keywords:** cremains weight, cremated remains, forensic anthropology, geographic variation, Italian population, sex dimorphism

## Abstract

The increased demand for the study of cremated remains, combined with their poor state of preservation, presents anthropologists with highly complex and challenging cases. In this context, cremains weight is considered a stable parameter, not influenced by the fragmentation state of the remains, useful in anthropological investigations. However, few data are available in the literature so far, and no study has been performed on the Italian population. To this purpose, the present study aims to provide cremains weights from a sample of 160 cremations belonging to Italian adult individuals, who were cremated at the Crematorium of Milan (Italy) recently (2012–2014). Mean weights were reported for both sexes, and the relationship with some anthropological and biological variables (i.e., age at death, height, body weight, and body mass index) was evaluated by univariate and multivariate analyses. As expected, the results showed a significant negative relationship with age at death (*p*‐value: <0.01) and a significant positive relationship with the remaining variables, especially in males (*p*‐value: <0.01). Comparison with the literature showed a close similarity with Portuguese data and a midway position between Asian and American samples, thus suggesting an intercontinental variation. However, few pieces of information are available so far to understand such variation, and further analyses are needed to identify the factors able to explain the variation observed. This is the first study supplying cremains weights for a middle‐aged and elderly Italian sample. This data could help anthropologists during the evaluation of human cremated remains by complementing or supporting other evidence.

**Highlights**

- Cremains weights for a recent Italian sample were examined.
- Biological and anthropological variables were included in the analyses.
- Comparisons with literature data suggest a geographical weight variation.

## 1 INTRODUCTION

Burned human remains represent one of the most challenging materials for anthropological analysis. The high degree of fragmentation, along with alterations such as color changes, shrinkage, warping, and fractures, significantly hinders personal identification and the determination of circumstances surrounding death [1, 2]. Furthermore, the mechanical reduction of the body to ash associated with cremation drastically limits the recovery of bone fragments with diagnostic value [3]. Some authors have proposed the use of alternative systems to derive identifying elements from such material, such as the search and identification of medical devices [4].

In this scenario, cremains weight is considered a stable parameter not influenced by the fragmentation rate of the burned remains [5]. Since the 1970s, studies on cremains collections have been conducted to provide reference values that support anthropologists in the analysis of cremated remains. Several authors have addressed the challenges of cremation, which may include incomplete recovery and commingling of remains. These issues can lead to inaccurate estimations of the minimum number of individuals (MNI) and, when possible, affect the reconstruction of the biological profile. These challenges highlight the importance of evaluating each context individually to assess the reliability of the data [3, 5, 6, 7, 8]. Small weights, in fact, are not necessarily exclusive to a single cremation; they may instead result from commingling not completely recovered. Additionally, inferences should consider weight variation by sex and age, as reduced weights are observed in females and in older subjects, and further anthropometric variables, such as body mass and height [5, 9, 10, 11, 12, 13].

The crucial need for reference data when analyzing the cremated remains recovered under suspicious circumstances highlighted the paucity of recent studies in the literature, and especially the lack of cremains weights for the Italian population. Most of the data available come from North America, specifically the United States (i.e., California [13, 14, 15], East Tennessee [10], Florida [9], and North Carolina [16]), followed by Europe (i.e., Poland [17], Germany [18], England [19], and Portugal [5]), and Asia (i.e., Thailand [11, 12]). Recent results have shown differences in cremains weights among data collected within the same geographic region, e.g., America and Europe [3, 5, 10, 13, 16]. Among the factors suggested to explain the variation observed, body weight and BMI (Body Mass Index) have been proven to be positively correlated with cremains weights, and to contribute to about 60% of the variation when considered with sex and age [3]. Since regional differences have been supposed [5, 10, 13], it is important to know the weight variation in the Italian population compared to the existing literature.

To this purpose, the present study aims to provide the cremains weights for a recent Italian sample and its relationship with biological/anthropometric variables, such as sex, age at death, height, and body weight. In addition, Italian results will be compared with those of European and non‐European populations.

## 2 MATERIALS AND METHODS

The study was conducted on 160 cremations belonging to Italian adult individuals performed at the Crematorium of Milan, Italy (Cemetery of Milano Lambrate), between 2012 and 2014. An agreement between the Municipality of Milan and LABANOF granted the latter permission to conduct observational studies at the crematorium on remains resulting from cremations. Only bodies in a good state of preservation and with a post‐mortem interval (PMI) lower than 72 hours were selected. Among these, 95.6% were autopsied (*n* = 153) and data concerning cause of death and recovery were available. In particular, 49% of the individuals died from natural causes, while in the remaining cases (51%) other causes of death were stated (i.e., domestic or traffic accidents, suicides, homicides, acute narcotism, medical responsibility). All the bodies were complete, and cases with limb amputation were not included in the sample.

The cremations were performed in methane gas ovens (G.E.M. Udine), in a single chamber. Bodies, inside a coffin and wearing garments, were placed in the chamber at the initial temperature of 600°C. During cremation, the temperature reached the mean maximum value of 1021°C (min: 853°C, max: 1155°C) in 30 min, and lasted on average 80 min (min: 65 min, max: 115 min). In the last step, the temperature gradually decreased until reaching 600°C; afterwards, the remains were collected. The analyses began in the minutes following the recovery.

Skeletal remains were manually separated from parts of the coffin (nails, metals, and carbon fragments), personal effects, and prostheses and were weighed using a digital scale.

The final stage of pulverization was performed at the end of the anthropological study.

The study was conducted in accordance with the Police Mortuary Rules.

### 2.1 Statistical analyses

Descriptive statistics included absolute frequencies (n) and percentages (%) for categorical variables, minimum (min), median, and interquartile range (IQR), maximum (max), as well as mean and standard deviations (SD) for continuous variables. We evaluated the relationship between the cremains weight (g) and age at death (years), height (cm), weight (kg), and BMI (kg/m^2^) of cadavers using univariable and multivariable linear regression models. All statistical tests were two‐sided with a significance level set at <0.05. Additionally, a weighted mean and a receiving operating characteristic (ROC) analysis were used to compute cut‐offs in cremains weight for discriminating sex.

All analyses were conducted using R version 4.0.5 (R Core Team, 2021).

## 3 RESULTS

Table 1 reports distributions of age at death, height, weight, BMI, and cremation weight of 160 cadavers according to sex. Among men (*n* = 106 cadavers), the mean ± SD of age at death was 62.6 ± 17.4 years, of height was 173.2 ± 7.7 cm, of weight was 80.8 ± 19.7 kg, of BMI was 26.9 ± 6.1 kg/m^2^, and of cremation weight was 3009.9 ± 561.8 g. Among women (*n* = 54 cadavers), the corresponding figures were 73.1 ± 13.0 years for age at death, 159.6 ± 7.3 cm for height, 66.2 ± 17.0 kg for weight, 26.3 ± 7.7 kg/m^2^ for BMI, and 2207.8 ± 542.2 g for cremation weight.

**TABLE 1:** Distribution of age at death, height, weight, BMI, and cremation weight of 160 cadavers according to sex.

Variable	Males (*n* = 106)				Females (*n* = 54)			
	Min	Median (IQR)	Max	Mean ± SD	Min	Median (IQR)	Max	Mean ± SD
Age at death (year)	21	64 (23)	97	62.6 ± 17.4	41	77 (17)	102	73.1 ± 13.0
Height (cm)^a^	154	173 (10)	193	173.2 ± 7.7	141	159 (9)	175	159.6 ± 7.3
Weight (kg)^b^	48.5	77.5 (22.4)	149.9	80.8 ± 19.7	41.0	62.0 (15.5)	126.8	66.2 ± 17.0
BMI (Kg/m^2^)^c^	15.3	26.0 (7.3)	50.7	26.9 ± 6.1	16.5	24.1 (6.5)	55.6	26.3 ± 7.7
Cremation weight (g)	1373	3051 (631)	5394	3009.9 ± 561.8	1268	2117 (541)	3576	2207.8 ± 542.2

^a^ One missing value.

^b^ Three missing values.

^c^ Four missing values.

Table 2 reports the sex classification of cadavers according to the optimal cut‐off of cremains weight obtained by the weighted mean (i.e., a weighted mean between means of cremains weight of men and women) and the ROC analysis. The cut‐off obtained by the weighted mean approach was 2739.2 g which correctly classified 75.6% of cadavers (69.8% of men and 87.0% of women). Using the ROC analysis, the optimal cut‐off was 2489.3 which correctly classified 82.5% of cadavers (84.0% of men and 79.6% of women).

**TABLE 2:** Sex classification according to the cut‐off of cremains weight obtained by the weighted mean and the receiver operating characteristics (ROC) analysis.

Method	*n*	Classification	
		Correct	Incorrect
		*n* (%)	*n* (%)
Weighted mean (cut‐off: 2739.2 g)			
Males	106	74 (69.8)	32 (30.2)
Females	54	47 (87.0)	7 (13.0)
Total	160	121 (75.6)	39 (24.4)
ROC (cut‐off: 2489.3 g)			
Males	106	89 (84.0)	17 (16.0)
Females	54	43 (79.6)	11 (20.4)
Total	160	132 (82.5)	28 (17.5)

**FIGURE 1:**
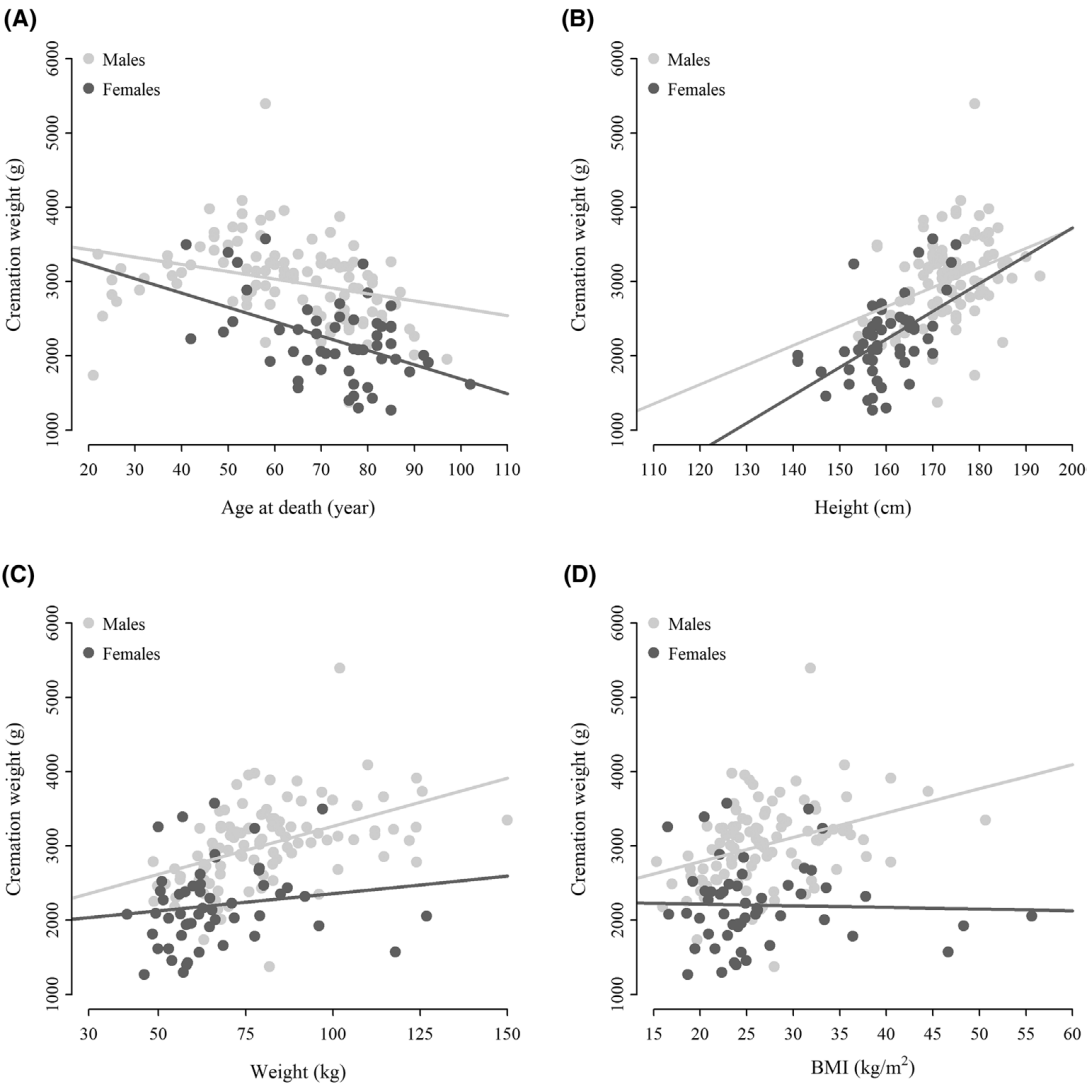
Scatter plots of cremation weights according to age at death (A), height (B), weight (C), and BMI (D) of cadavers. Light and dark gray points are the weights of cremated remains for men and women, respectively.

Figure 1 shows the scatter plot of cremains weight according to age at death, height, weight, and BMI of cadavers. The cremains weight decreased with increasing age at death for both males and females (A). Conversely, the cremains weight increased with increasing height for both sexes (B). Likewise, positive relationships between cremains weight and weight of cadavers (C) and between cremains weight and BMI of cadavers (D) were observed only for men. Quantitatively, a 1‐year increment in the age at death of the cadavers corresponded to a significant decrease of cremains weight of 9.85 g (95% CI: −15.82, −3.87; *p* < 0.01) and of 19.34 g (95% CI: −29.65, −9.03; *p* < 0.01) for males and females, respectively (univariable analyses in Table 3). An increment of 1 cm in the height of cadavers resulted in a significant increase of cremains weight of 26.15 g (95% CI: 12.86, 39.45; *p* < 0.01) for males and of 37.58 g (95% CI: 19.86, 55.31; *p* < 0.01) for females. For men, a 1‐unit increment in the weight and the BMI of cadavers significantly increased the cremains weight of 12.96 g (95% CI: 8.04, 17.89; *p* < 0.01) and of 32.71 g (95% CI: 15.80, 49.63; *p* < 0.01), respectively. For women, the weight and the BMI were not associated with the cremains weight (*p* = 0.30 and *p* = 0.83, respectively).

**TABLE 3:** Sex‐specific univariable and multivariable linear regression models for cremains weight according to age at death, height, weight, and BMI of cadavers.

Variable	Univariable		Multivariable^a^	
	β (95% CI)	Wald test	β (95% CI)	Wald test
Age at death (year)				
Males	−9.85 (−15.82, −3.87)	*p* < 0.01	−5.40 (−11.09, 0.29)	*p* = 0.06
Females	−19.34 (−29.65, −9.03)	*p* < 0.01	−12.44 (−23.42, −1.46)	*p* = 0.03
Height (cm)^b^				
Males	26.15 (12.86, 39.45)	*p* < 0.01	13.59 (0.03, 27.15)	*p* = 0.05
Females	37.58 (19.86, 55.31)	*p* < 0.01	30.17 (11.09, 49.25)	*p* < 0.01
Weight (kg)^c^				
Males	12.96 (8.04, 17.89)	*p* < 0.01	10.11 (4.93, 15.29)	*p* < 0.01
Females	4.65 (−4.31, 13.61)	*p* = 0.30	3.04 (−4.73, 10.80)	*p* = 0.44
BMI (kg/m^2^)^d^				
Males	32.71 (15.80, 49.63)	*p* < 0.01	–	–
Females	−2.21 (−22.27, 17.86)	*p* = 0.83	–	–

^a^ Included age, height, and weight of cadavers.

^b^ One missing value.

^c^ Three missing values.

^d^ Four missing values.

In the multivariable analysis, significant relationships between cremains weight and age at death (−12.44; 95% CI: −23.42, −1.46; *p* = 0.03) and cremains weight and height (30.17; 95% CI: 11.09, 49.25; *p* < 0.01) emerged for females. Whereas the multivariable analysis showed a significant relationship between cremains weight and weight of cadavers (10.11; 95% CI: 4.93, 15.29; *p* < 0.01), and relationships at the limit of significance between cremains weight and age at death (−5.40; 95% CI: −11.09, 0.29; *p* = 0.06) and cremains weight and height (13.59; 95% CI: 0.03, 27.15; *p* = 0.05) for males.

## 4 DISCUSSION

The present study provided an insight into how cremains weight varies according to some biological and anthropological variables (i.e., sex, age at death, height and cadaver weight) within a contemporary Italian sample. In particular, our multivariable analysis showed a significant negative association between cremains weight and age at death for females and a relationship at the limit of significance for males. In addition, the height of cadavers was significantly positively associated with cremains weight in both sexes, while a significantly positive relationship between the weight of cadavers and cremains weight was detected only in males. A sectioning point for sex estimation was also calculated by weighted mean and ROC analysis, providing considerable accuracies. However, as already reported, caution should be used when inferring sex in unknown circumstances, as other factors could cause changes in skeletal weight (e.g., increasing age and some pathological conditions) [3, 5, 13].

Previous research has explored weight variation mainly in relation to sex and age at death of cadavers [5, 10, 13, 16, 18, 19]. Only two studies also considered the cadavers height and weight [9, 11, 12], but none were performed on European populations.

**FIGURE 2:**
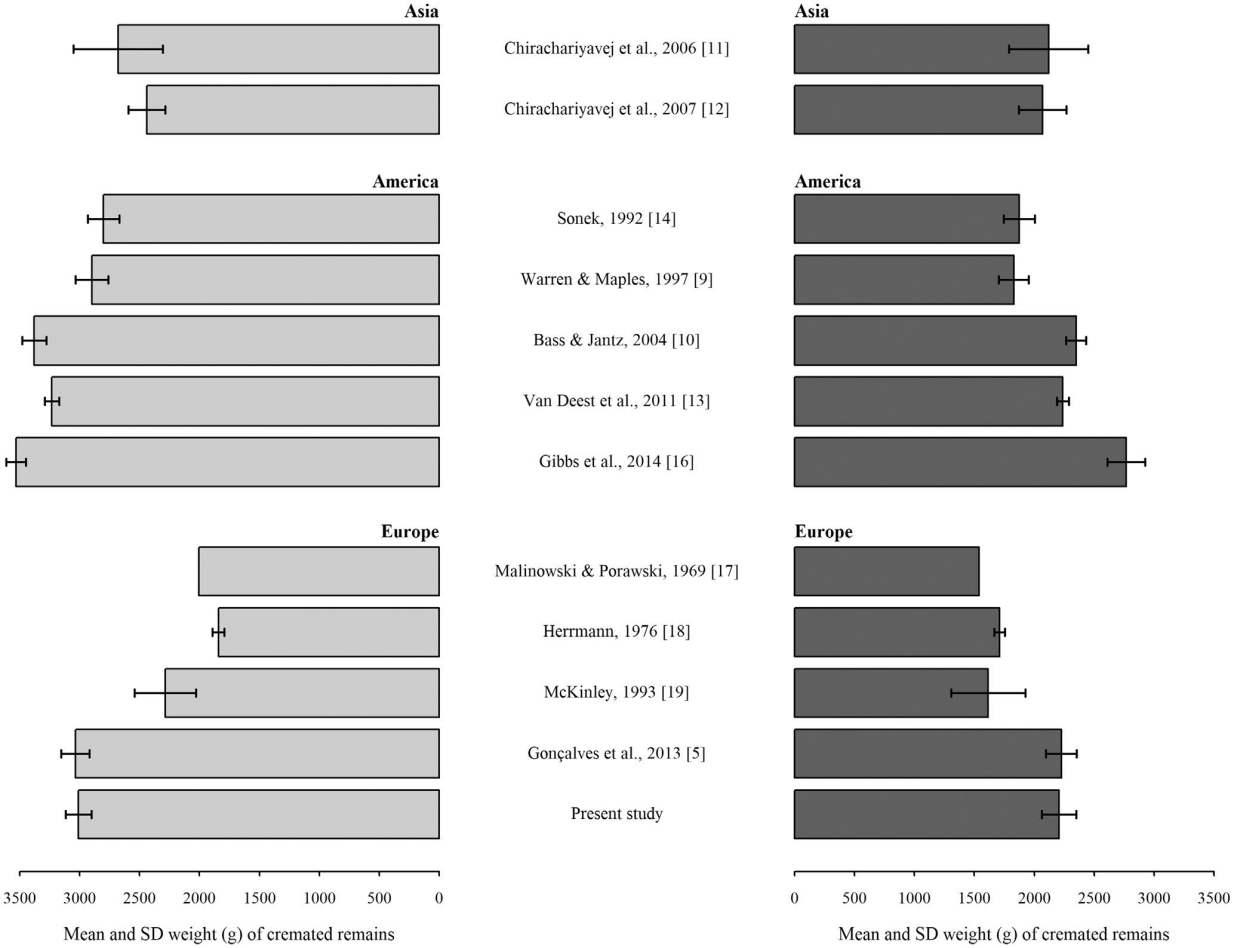
Mean and standard deviation weight of cremated remains across different studies. Light and dark gray bars are the weight of cremated remains for men and women, respectively. Standard deviations were not reported in Malinowski and Porawski's study.

As expected, weight differences between the sexes were detected: Italian male cremations were on average c. 800 g heavier than females (see Figure 2), in agreement with the most recent published studies, which showed differences ranged between ca. 765 g and 1030 g [5, 10, 13, 16]. This difference was particularly evident when the cremains weight was related to age at death in both sexes separately. The significant negative relationship observed was more skewed towards the older ages in females than in males, showing a decrease in cremains weight of about twice the rate of males. A similar decreasing rate was observed in Bass and Jantz [10], who ascribed the weight loss to the bone loss recorded with age, and especially to the accelerated bone loss recorded in females during menopause, well‐known in the literature [20, 21]. Age‐related bone loss is generally linked to bone tissue homeostasis alteration, and specifically to the imbalance between bone formation and resorption, particularly evident in aged individuals [22, 23]. Sex hormone deficiency is one of the main causes, but other causes may lie in mineral intake, pathologies (e.g., rheumatoid arthritis, gastrointestinal disorders causing malabsorption, and myeloma), drugs (such as corticosteroids), and behavioral factors (i.e., physical activity, smoking, and alcohol consumption) [24].

Similarly, the positive relationship detected between cadaveric stature and weight and cremains weight was expected, confirming previous findings [3, 9, 12]. However, in contrast to [3, 9, 12], only the male sample showed a significant relationship between cadaveric weight (or BMI) and cremains weight. These results could be ascribed to the small size of the female samples (two times smaller than males) and to the low variability observed within the female data, which appeared gathered on the left of the scatter plots (Figure 1C,D).

Literature data on cremains are not always homogenous: differences in sample composition and size are evident, and some information, especially about the cremation process (i.e., container used, temperature and duration of cremation), is lacking, making weights at times hardly comparable, in particular with older studies (Table 4).

**TABLE 4:** Characteristics of study population, characteristics of cremation, and age at death according to sex of cremated cadavers across different studies.

Author, year	Population characteristics			Cremation characteristics			Age at death, years	
	Country/State	Sample	Period	Container	Temperature	Duration	Males	Females
			Century		°C	Min	Mean ± SD; range	Mean ± SD; range
Asia								
Chirachariyavej et al. (2006) [11]	Thailand	Cadavers	21th	Particleboard	850–1200	60–90	63.5 ± 18.9; 9–107	73.3 ± 17.3; 9–107
Chirachariyavej et al. (2007) [12]	Thailand	Cadavers	21th	Particleboard	850–1200	60–90	59.6 ± 17.8; 9–107	69.2 ± 18.0; 9–107
America								
Sonek (1992) [14]	California	Suspected cadavers	20th	Not reported	Not reported	Not reported	64.1	75.7
Warren and Maples (1997) [9]	Florida	Cadavers	20th	Cardboard	>830	73–207	66.3 ± 14.0; 25–97	74.1 ± 11.0; 50–91
Bass and Jantz (2004) [10]	East Tennessee^a^	Cadavers	21th	Different	871–982	120–180	62.8 ± 17.2; 18–99	70.7 ± 16.8; 19–98
Van Deest et al. (2011) [13]	California	Suspected cadavers	21th	Cardboard or other	871–927	120	71.4 ± 16.9; 20–102	76.1 ± 15.3; 20–106
Gibbs et al. (2014) [16]	North Carolina	Suspected cadavers	21th	Not reported	Not reported	Not reported	19‐ ≥ 66	19‐ ≥ 66
Europe								
Malinowski and Porawski (1969) [17]	Poland	Mean of bone categories	20th	Not reported	~1000	Not reported	45–65	45–65
Herrmann (1976) [18]	Germany	Cadavers	20th	Not reported	Not reported	Not reported	72.8 ± 13.3	76.2 ± 10.2
McKinley (1993) [19]	England	Cadavers	20th	Not reported	Not reported	Not reported	77.3 ± 10.0; 66–90	81.7 ± 10.8; 62–94
Gonçalves et al. (2013) [5]	Portugal	Cadavers	21th	Wooden coffin	750–1050	60–180	68.6 ± 14.8; 34–93	74.5 ± 15.1; 41–97
Present study	Italy	Cadavers	21th	Different	853–1155^b^	65–115^b^	62.6 ± 17.4; 21–97	73.1 ± 13.0; 41–102

^a^ American white ethnicity.

^b^ Available for 86 samples (74 missing values).

Our cremains sample showed weights greater than those observed in 20th century data from both European and American populations, the former with differences greater than the latter (weight difference in the European sample: 496 g – 1168 g; weight difference in the American sample: 111 g – 378 g). At first glance, this may be related to changes in the individual skeletal mass due to, for example, increasing stature over time, or to possible different cremation processes adopted in the two different periods. However, previous data excluded secular trend as the main factor influencing cremains weights [13], and missing data about the cremation process prevented any consideration of the combustion process. Moreover, looking at the sample composition, the observed differences may be firstly related to differences in the samples analyzed rather than regional or temporal influence. This is especially true for European data. In fact, the small sample size and the older age of the individuals may have underestimated the general weight of the English population (sample size equal overall to 15 individuals, and age at death greater than 60 years old), and the different cremains weight calculation for the Polish sample, which was carried out by adding the mean weights of the different bone categories considered, could have made these results not comparable. Differently, for the German sample differences may be ascribed to factors that cannot be identified exactly with the information available, even though the mean age of the sample, slightly older, and, in general, the little sexual dimorphism detected, in contrast to all current literature, suggested some differences in sample composition, as already highlighted by [5].

Differences were also observed with recent cremation studies. A peculiar trend was in fact detected: our cremains weights were very close to the Portuguese sample and both were heavier than the Thai and lighter than the American samples (Figure 2). This difference was more pronounced for the male cremains sample. An intercontinental variation could be suggested for the American and European populations due to the similar characteristics of the samples studied (especially age at death). Conversely, the lower cremains weights detected in the Thai population may be a reflection of a geographic variation or a confounding effect due to the inclusion of children in the study (range of age at death: 9–107). However, the similar mean age at death between the Thai and our samples may be indicative of quite comparable data, thus suggesting intercontinental variation as one of the likely variables involved in such cremains weight difference.

**FIGURE 3:**
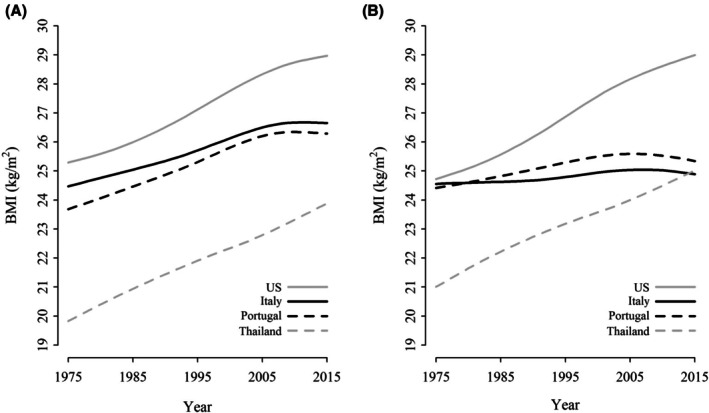
Mean BMI distribution for men (A) and women (B) according to geographical area from 1975 to 2015. Data on BMI are available at the NCD Risk Factor Collaboration, NCD‐RisC, website [www.ncdrisc.org].

These observations corroborated the previous hypothesis of a geographical weight variation [5, 10, 13]. To date, no sufficient data are available to identify the factors responsible for this evidence. Body mass is one of the most considered [3, 10, 13]. In fact, variation in diet, activity, and bone mineral density may affect the individual mass, and consequently the skeletal mass, resulting in different weights after cremation [13]. This hypothesis could be supported by the peculiar (and similar) trend observed when the BMI values of the four populations (America, Portugal, Italy, and Thailand) were compared (BMI values from 1975 to 2015 available at the NCD Risk Factor Collaboration, NCD‐RisC, website [www.ncdrisc.org]): Portuguese and Italian BMIs were, in fact, very close to each other, and both halfway between the Thai and American populations, the former showing the lowest values (see Figure 3). However, BMI is not the only variable involved in cremains weight variation. In fact, in conjunction with sex and age, BMI was not able to explain the entire variability observed [3].

Bone mineral density (BMD) may represent another factor positively associated with cremains weight. To date, no studies have compared BMD values to the weights of skeletal remains after cremation. Population data highlighted a significant difference in BMD between individuals of Asian ancestry and White Americans (the former with values lower than the latter) [25, 26, 27], thus providing a further possible explanation for the low cremains weights detected in the Thai population. Differently, few and contrasting data are available on European and American populations [26, 28, 29, 30], and especially on samples whose composition was comparable with cremation studies. Therefore, even though BMD could be suggested as a putative factor influencing cremains weights, the lack of sufficient population data prevents, at the moment, any inference on its involvement in cremains weight variation.

By comparing our data with the literature, another peculiar pattern emerged: the female sample, in fact, showed smaller differences than the male counterpart, especially when the Thai, Tennessee, and California samples were considered. However, few pieces of information are available at the moment to identify the possible causes of this variation. The scarce knowledge of the factors involved in cremains weight variation, the small size of our female sample (two times less than male), and the lack of data (such as pathology, drug consumption, behavioral factors) about all the samples analyzed, in fact, have made any consideration difficult. In addition, data were collected from different countries in different years, thus changes could have occurred over time, as reported for BMD in the US population [31, 32, 33], leading to differences in the samples considered. Further analyses are therefore needed to understand the mechanisms underlying the variation observed in cremains weights and in the different population groups.

The present study has some limitations. First of all, the small size of the female sample compared to the male counterpart and, in particular, the few numbers of young individuals. This is related to uncontrollable factors such as the death rate by sex and age and the request for cremation rather than burial of the body. However, despite the different sample size, the study was able to identify significant differences between males and females, providing data on cremains weight for the middle‐aged adults and elderly classes. An additional limitation lies in the lack of information about the medical history of the individuals analyzed (e.g., pathologies, pharmacological treatments, habits), which prevented conducting stratification analyses, especially by age‐group categories. Finally, the limited homogeneity between our data and the literature studies available, and the few information on the combustion process adopted. Even if there was no certainty that the samples were fully comparable, as already reported by [5], considering the differences observed, especially among the different continents, a real geographical variability may be considered.

It should be noted that this data comes from an observational study at the crematorium aimed at analyzing remains resulting from cremations before the pulverization step, as reported in [34] for fetal bones; thus, the weighing procedure was performed before processing remains into ashes, differently from the referenced studies. However, as reported in [5], the fragmentation rate does not influence the weight; for this reason, the timing of weighing should not have affected the results obtained, and the cremains weights can be compared to those of the other studies.

In conclusion, this study represents the first one which provides cremains weights for a recent Italian sample and evaluates its relationship with some biological and anthropological variables, comparing data with other European and non‐European samples. The continental variation identified highlights the importance of having data from different population groups in order to support the anthropologists who face the analysis of cremated remains. For this reason, further analyses are needed to increase the evidence (e.g., including also the younger population) in order to have a comprehensive framework on the factors underlying variation in the cremains weights in the Italian population. In the meantime, data obtained in this study summarize the cremains weights for an Italian male and female sample, composed especially of middle‐aged adults and elderly. The data may be useful in forensic investigations, to complement or to support other evidence.

## CONFLICT OF INTEREST STATEMENT

We declare to have no conflict of interests and to have had no financial support for the current study.

## Data Availability

Data are available from the corresponding author upon reasonable request.
